# Cystic Lesions and Odontogenic Tumors in Older People: A Brazilian Multicenter Study

**DOI:** 10.4317/jced.60658

**Published:** 2024-10-01

**Authors:** John Lennon Silva Cunha, Sarah dos Santos Martins, Elton Fernandes Barros, Israel Leal Cavalcante, Caio César da Silva Barros, Eveline Turatti, Roberta Barroso Cavalcante, Felipe Paiva Fonseca, Pollianna Muniz Alves, Cassiano Francisco Weege Nonaka, Bruno Augusto Benevenuto de Andrade

**Affiliations:** 1 Department of Oral Diagnosis, School of Dentistry of Piracicaba, State University of Campinas (UNICAMP), Piracicaba, Brazil; 2 Center for Biological and Health Sciences, Federal University of Western Bahia (UFOB), Barreiras, Bahia, Brazil; 3 Department of Dentistry, State University of Paraíba (UEPB), Campina Grande, Brazil; 4 Department of Oral Diagnosis and Pathology, School of Dentistry, Federal University of Rio de Janeiro (UFRJ), Rio de Janeiro, Brazil; 5 Postgraduate Program in Dental Sciences, Department of Dentistry, Federal University of Rio Grande do Norte (UFRN), Natal, Brazil; 6 Department of Dentistry, University of Fortaleza (UNIFOR), Fortaleza, Brazil; 7 Department of Clinical, Pathology and Dental Surgery, School of Dentistry, Federal University of Minas Gerais (UFMG), Belo Horizonte, Brazil

## Abstract

**Background:**

Some odontogenic cysts (OCs) and odontogenic tumors (OTs) are infiltrative and often recur, causing bone destruction and tooth loss. In the elderly, in particular, these injuries cause significant morbidity, making rehabilitation difficult and compromising the quality of life of these individuals. Objective: To determine the prevalence and demographic characteristics of OCs, non-odontogenic cysts (NOCs), and OTs diagnosed in an elderly Brazilian population (≥60 years).

**Material and Methods:**

A retrospective descriptive cross-sectional study was carried out in three Brazilian pathology referral centers (1999-2019). Data regarding age, sex, ethnicity, anatomical location, symptomatology, and histopathological diagnosis were obtained from histopathological records and analyzed. Pearson’s Chi-squared and Fisher’s exact tests were used to evaluate the association between the different groups of oral lesions and demographic findings, adopting a P-value of ≤ 0.05 and a 95% confidence interval.

**Results:**

A total of 7,476 histopathological records were evaluated, of which 389 (5.2%) cases were classified as OCs, 86 (1.15%) as NOCs, and 83 (1.11%) as OTs. The most common lesions in each group were periapical cysts (n=166; 68.9%), ameloblastomas (n=65; 77.4%), and salivary duct cysts (n=45; 52.3%). Overall, males were slightly more affected (n=279, 50.2%). Most individuals were between 60 and 69 years (n=358; 64.2%). OCs and OTs preferentially affected the mandible (n=280; 62.2%). NOCs occurred more frequently in the lips (n=19; 22.1%), followed by buccal mucosa (n=18; 20.9%). The overall concordance between clinical and histopathologic diagnoses was 47.2% (213 of 451 cases).

**Conclusions:**

OCs were relatively common, whereas NOCs and OTs were rarer among the elderly. The low concordance between clinical and histopathological diagnosis highlights the importance of histopathological analysis to ensure an accurate diagnosis. Dentists and geriatricians must be familiar with these lesions to ensure an early diagnosis, reduce morbidity and improve the quality of life of these individuals.

** Key words:**Odontogenic cysts, Non-odontogenic cysts, Odontogenic tumors, Oral diseases, Older people, Oral lesions.

## Introduction

The segment of the elderly population is growing worldwide, more than any other age group (1-4). Population aging is followed by a high prevalence of oral and systemic diseases (1-3), which will increase the demand for oral medicine services (2). This modification in the demographic profile of the population imposes new challenges on health services, especially those related to oral health, because most older people have precarious oral health, resulting from a healthcare model that, for many years, favored mutilating and curative dental practices (dental restorations and extractions) rather than prevention strategies (1,2). Therefore, knowing the most prevalent oral lesions in this population through histopathological studies is essential for geriatricians and dentists as it provides accurate information on the profile of these lesions in the elderly, contributing to early diagnosis (1,3,5).

OCs and OTs occur mainly in the jaws of individuals between the second and fourth decades of life (6). In most cases, these lesions have an indolent behavior. However, some OCs and OTs, such as odontogenic keratocysts (OKCs) and ameloblastomas, have a locally invasive behavior and frequently recur, causing considerable bone destruction and tooth loss (3,6). Also, most malignant OTs have aggressive nature, metastatic potential, and a poor prognosis (5). In the elderly, specifically, these lesions tend to cause significant morbidity, making oral rehabilitation challenging and considerably decreasing the quality of life of these individuals (3,5). On the other hand, NOCs generally have indolent clinical behavior. They can be located either in soft tissues or intraosseous sites, such as the maxilla and mandible and rarely recur and cause extensive bone destruction (3,7,8).

Although several studies have reported the prevalence and incidence of OTs and cystic lesions in the oral and maxillofacial region, only a few have characterized the lesions according to age group (9-11). This analysis is essential because it facilitates understanding the most common lesions in each age group, guiding clinicians in the correct diagnosis (1,3). In addition, they provide valuable information that supports the development of appropriate preventive and therapeutic measures. Therefore, this study aimed to evaluate the prevalence and main demographic characteristics of OCs, NOCs, and OTs in a population of elderly Brazilians population (≥ 60 years).

## Material and Methods

-Study design and sample 

**Figure 1:**
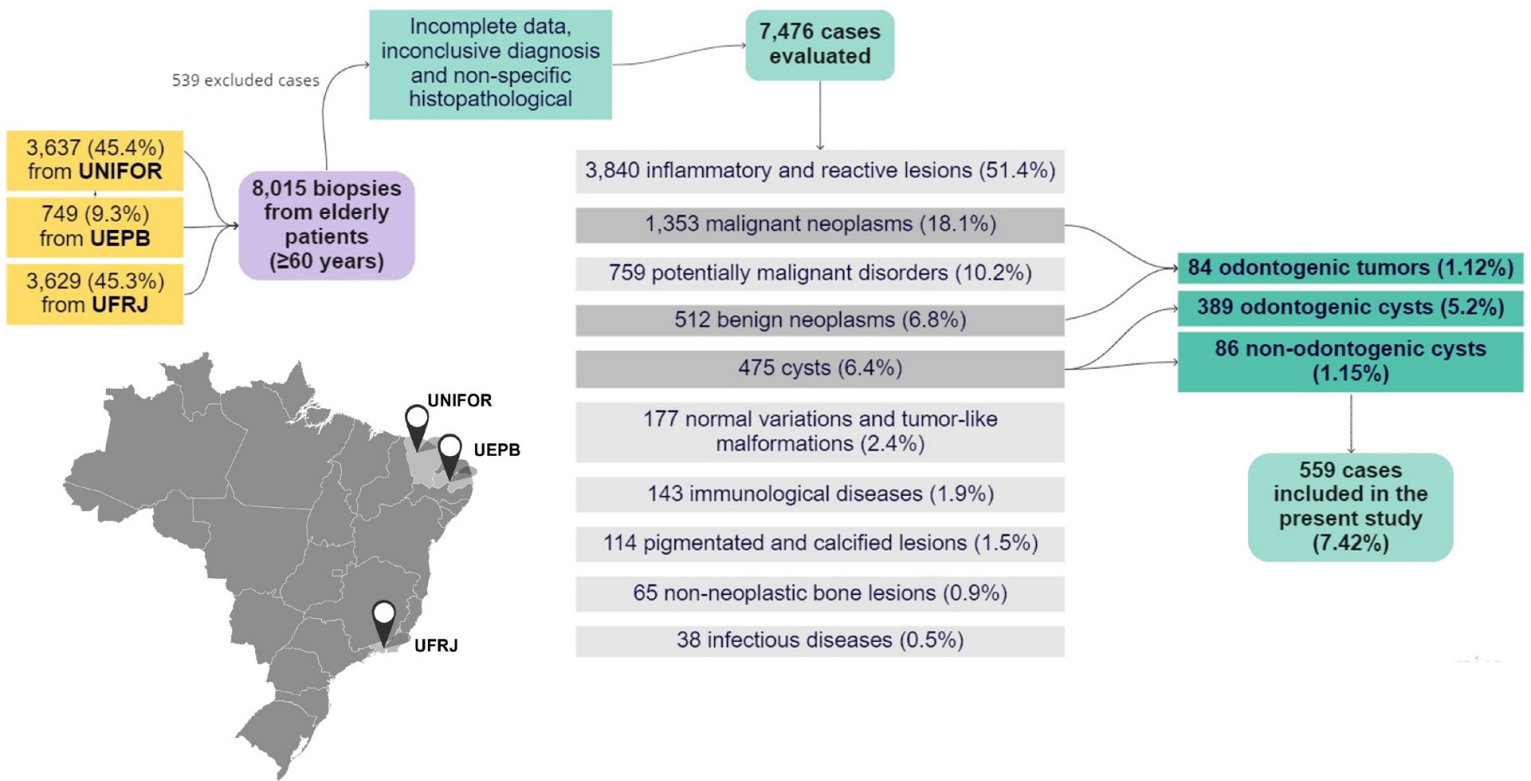
Flowchart showing the sample selection from the three oral pathology centers participating in the study. The primary data were published by Cunha et al. (1), and the present study focused on the detailed analysis of cystic lesions and odontogenic tumors in older people.

In this multi-institutional retrospective study (1999-2019), the histopathological records of all older people (≥60 years old) diagnosed with OCs, NOCs, and OTs in the oral and maxillofacial region were retrieved from the archives of three Brazilian oral pathology services (Fig. 1). Data such as age, sex, ethnicity, anatomical site, symptomatology, and histopathological diagnosis were collected from biopsy records and analyzed. The lesions were categorized into OCs, NOCs, and OTs according to the 5th edition of the World Health Organization (WHO) Classification of Head and Neck Tumours (2022) (12). The cysts not included in the current classification were classified according to previous literature (3).

This multi-institutional retrospective study is part of a previous analysis carried out by our research group, which analyzed 7,476 cases of oral and maxillofacial lesions in older people (≥ 60 years) (1).

This study was approved by the Ethics Committee of the State University of Paraíba (UEPB) (CAAE: 61639722.9.0000.5187).

-Data analysis

Data were subjected to descriptive and quantitative analysis using the Statistical Package for the Social Sciences (SPSS) for Windows 20.0 (SPSS. Inc., Chicago, IL, USA). Continuous variables were expressed as mean, median, and standard deviation values (SD). Categorical variables were defined as the absolute number of cases and percentage values. Fisher’s exact and Chi-square tests were used to assess the association between the different groups of oral lesions and demographic characteristics, adopting a P-value of ≤ 0.05 and a 95% confidence interval.

## Results

A total of 7,476 cases of oral and maxillofacial lesions were diagnosed in the elderly (≥ 60 years) at the three Brazilian centers participating in the study (1999-2019), of which 389 (5.2%) were OCs, 86 (1.15%) NOCs, and 84 (1.12%) OTs (Fig. 1). This study provides detailed features of these lesions.

Overall, males were slightly more affected (n = 279, 50.2%). Most individuals were aged between 60 and 69 years (n = 358; 64.2%), with few cases over 80 years (n = 50; 8.9%). A statistically significant association was observed between the age of the individuals (60-69 years) and the lesion groups (odontogenic cysts) (*P* = 0.0011). OCs were the most common lesions (n = 389; 69.7%), followed by NOCs (n = 86; 15.3%) and OTs (n = 84; 15.0%) ( Table 1).

Regarding OCs, males (n = 211; 54.7%) were more affected than females (n = 175; 45.3%), with a male-to-female ratio of 1.2:1; however, there was no statistically significant association ( Table 2). Inflammatory odontogenic cysts were more common (n = 241; 62.0%) than developmental odontogenic cysts (n = 148; 38.0%). Among those of an inflammatory nature, the most common were periapical cysts (n = 166; 68.9%) and residual cysts (n = 68; 28.2%). Concerning developmental cysts, the most common cyst was OKC (n = 68; 45.9%), followed by odontogenic cyst not otherwise specified (n = 37; 25.0%), and dentigerous cyst (n = 19; 12.8%). Other cysts such as calcifying odontogenic cyst (n = 5; 3.4%), adult gingival cyst (n = 4; 2.7%), lateral periodontal cyst (n = 3; 2.0%), and orthokeratinized odontogenic cyst (n = 2; 1.4%) were highly uncommon in this population.

**Figure 2:**
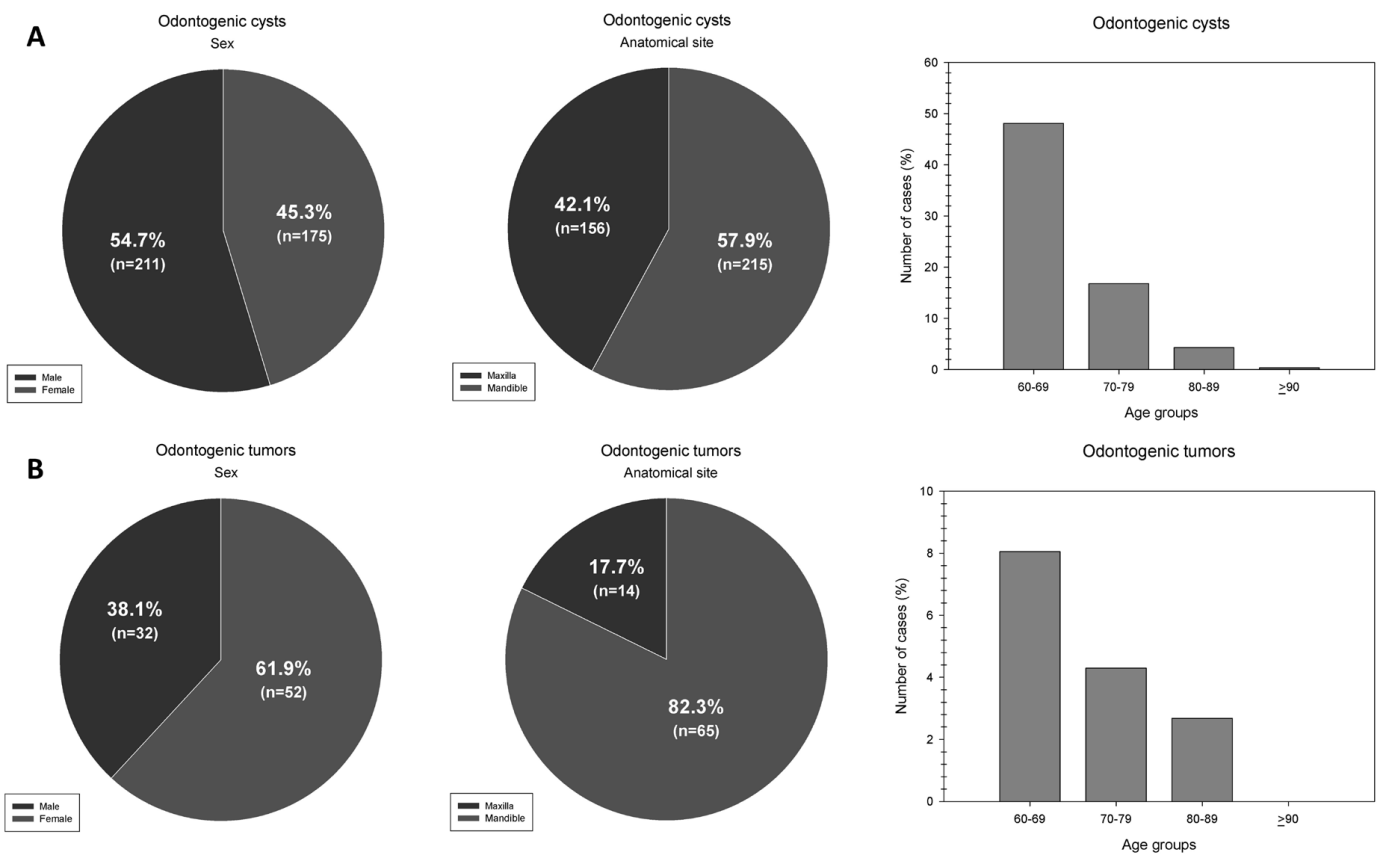
Age group (decade of life), sex, and anatomical site distribution of (A) odontogenic cysts and (B) odontogenic tumors in the elderly.

The mandible was the most affected anatomical location (n = 215; 57.9%), mainly in the developmental cyst group. Inflammatory odontogenic cysts were more common in the maxilla when compared to the mandible (*P* = 0.0008) (Fig. 2). Signs and symptoms, such as pain and swelling, were reported in 167 (42.9%) odontogenic cysts, while 131 cysts (33.7%) were asymptomatic. No signs or symptoms were described in 91 cases (23.4%). The pain was significantly more present in older adults with inflammatory cysts (n = 104; 62.3%) than those with developmental cysts (n = 31; 18.6%) (*P* < 0.001).

Regarding odontogenic neoplasms, most tumors were benign (n = 83; 98.8%). Only one malignant odontogenic tumor was diagnosed in this population (1.2%). Overall, OTs occurred mainly in the mandible (n = 65; 82.3%) of women (n = 52; 61.9%), with a female-to-male ratio of 1.6:1 (Fig. 2). Ameloblastoma was the most common benign tumor (n = 65; 77.4%), and the rarest was the squamous odontogenic tumor (n = 1; 1.2%) ( Table 3). Signs and symptoms, such as swelling and pain, were reported in 41.7% of cases (n = 35), while 23 (27.4%) cases were asymptomatic. In 26 cases, signs/symptoms were not reported (31.0%).

**Figure 3:**
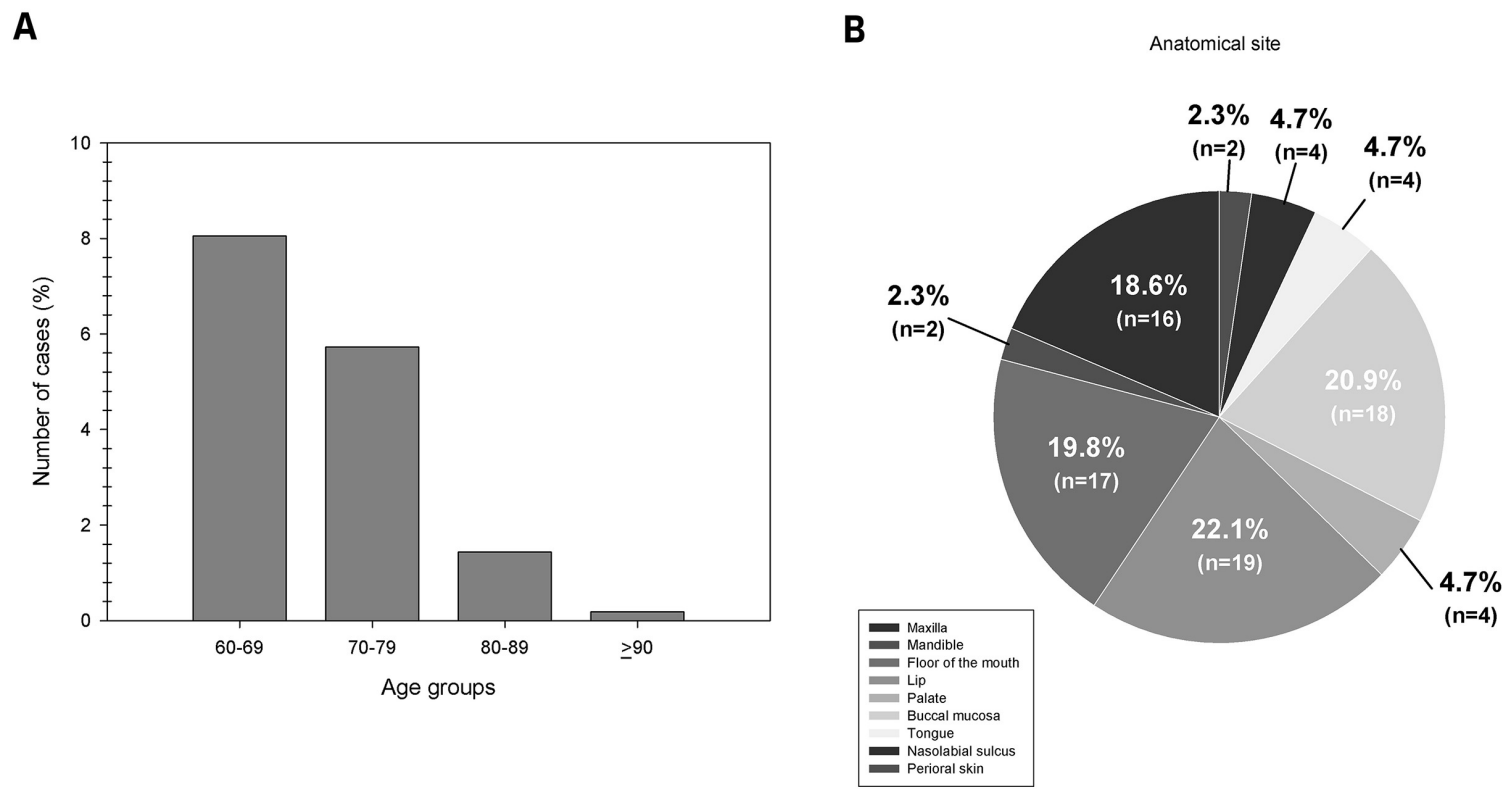
(A) Age group (decade of life), and (B) anatomical site distribution of non-odontogenic cysts in the elderly.

NOCs occurred mainly in women (n = 50; 58.1%) with a female-to-male ratio of 1.4:1. The lips (n = 19; 22.1%) were the most affected anatomical location, followed by buccal mucosa (n = 18; 20.9%) and floor of the mouth (n = 17; 19.8%). On the other hand, the maxilla was the most affected intraosseous site (n = 16; 18.6%) (Fig. 3). The most common NOCs were salivary duct cysts (n = 45; 52.3%), followed by nasopalatine duct cysts (n = 15; 17.4%) and epidermoid cysts (n = 8; 9.3%) (Table 4). As for symptoms, most cases were asymptomatic (n = 47; 54.7%), 13 (15.1%) reported pain, and in 26 cases (30.2%), this information was not available.

The overall concordance between clinical and histopathologic diagnoses was 47.2% (213 of 451 cases). The highest agreement was observed in the group of OCs (53.5%), followed by OTs (36.8%) and NOCs (25.4%).

## Discussion

Herein, we report the demographic characteristics of 558 cystic lesions and OTs in older people diagnosed at three Brazilian oral and maxillofacial pathology services, from a total of 7,476 diagnostics. Of these, 389 (5.2%) were diagnosed as OCs, 86 (1.15%) as NOCs, and 84 (1.12%) as OTs, similar to previously reported prevalence rates (3,13).

By definition, a cyst is a pathological cavity covered by epithelium, often filled with liquid or semi-solid material. Various cysts can arise in the oral and maxillofacial region (3,12). In the 2017 WHO Classification of Head and Neck Tumours, the jaw cysts were separated into two groups: (i) inflammatory OCs and (ii) odontogenic/non-odontogenic developmental cysts. In the current WHO classification (2022), the umbrella term ‘cysts of the jaws’ was used without any subdivision (12). Nevertheless, herein, we have discussed them under the subheadings of NOCs and OCs to emphasize their origin (12). In the current study, the prevalence of OCs was four times greater than NOCs, slightly smaller than that observed in a previous study among the elderly (3), but similar to the general population (9,10,14,15).

NOCs were uncommon in the present study, accounting for only 1.11% of all lesions diagnosed in the elderly. However, these lesions are uncommon in all age groups (3,9-11,14). In our study, salivary duct cyst was the most commonly seen NOCs in the elderly, followed by nasopalatine duct cyst (NDC), similar to previous reports (3). However, some studies have shown the nasopalatine duct cyst as the most prevalent NOC in the elderly and the general population (8-10,13,15). Regarding the anatomical site, most soft tissue cysts occurred in the lips, buccal mucosa, and the floor of the mouth, similar to previous studies (7,8). On the other hand, as previously reported, most cases of intraosseous NOCs were located in the maxilla (3,7-11,14,16). Overall, conservative surgical excision effectively treats these lesions. NOCs have low recurrence rates and an excellent prognosis (3).

Inflammatory odontogenic cysts correspond to about 38 to 60% of all cysts and commonly occur in adults and the elderly; lesions in children and adolescents are uncommon (15). In the present study, the inflammatory cysts were the most common in older people, with the periapical and residual cysts as the most prevalent. Although residual cysts have been included in the periapical cyst diagnosis in the 2017 WHO classification (17), we addressed them separately for didactic reasons. If analyzed separately, the residual cyst corresponded to the 2nd most common lesion in our sample. The development of periapical and residual cysts occurs due to inflammatory and degenerative changes in the dental pulp, often leading to tooth loss (3). The higher prevalence of these cysts has been associated with poor oral health conditions and lower socioeconomic status (18,19), which emphasizes the importance of developing and encouraging preventive oral health policies to ensure better oral health conditions in this population worldwide (6). When not appropriately treated, these lesions can reach large sizes and involve adjacent teeth (3). Therefore, an accurate early diagnosis and adequate therapeutic approach are essential to prevent loss of teeth and bone support, facilitating oral rehabilitation and improving masticatory efficiency and quality of life.

Regarding developmental odontogenic cysts, the most common in the elderly was OKC (17.5%). Contrary to previous studies, dentigerous cysts are often the most prevalent developmental cysts, especially among young people in the second and third decades of life (9,11,12,15,16,20-22). As they arise due to fluid accumulation between the crown of the unerupted tooth and the reduced enamel epithelium, usually impacted lower third molars and upper canines, the low prevalence of these cysts in the elderly is not surprising. On the other hand, since OKC has a high infiltrative capacity and recurrence rates, its treatment typically involves aggressive surgical approaches, which cause significant bone loss and tooth loss (3,23).

Although these findings have already led the WHO to classify the OKC as an odontogenic tumor of epithelial origin, it was reclassified as a developmental odontogenic cyst in 2017 (12,17). Most OKCs show PTCH1 gene mutations but rarely PTCH2 or SUNU mutations (12). Although the incidence and prevalence of OCs and OTs vary according to the type of classification used for the lesions and the geographic location of the study, the OKCs are usually the third most common cysts in the general population. However, they are usually less frequent in elderly patients (≥60 years) (3).

Regarding the anatomical site, OCs occurred mainly in the mandible (n = 215; 57.9%). Accordingly, previous studies on the elderly have shown that the mandible is the most affected anatomical location, responsible for more than 50% of all cases (3). These data indicate that the preferred anatomical site of these cysts does not vary according to age group. However, in the present investigation, OCs were more common in older men, unlike previous reports, which showed a higher prevalence in women (3). On the other hand, NOCs were slightly more frequent in women. It has been suggested that the higher prevalence of oral lesions in women may result from a greater concern for oral health than in men (3). Perhaps women seek more health services when needed; therefore, some of these lesions are diagnosed more frequently in this population (3).

The prevalence of OTs in the present study was low (1.11%) but similar to another Brazilian survey of oral lesions in the elderly (3). OTs are more common in young people between the second and fourth decades of life, with a small proportion of cases appearing in the elderly (11,14,16,24-27). In the current study, the most common odontogenic tumor was ameloblastoma (77.4%), but many other subtypes occurred in the elderly population studied ( Table 3). Despite this variety of histological subtypes, the most clinically significant tumors are ameloblastomas. These neoplasms have a potential for bone destruction and high recurrence rates, causing major aesthetic and functional complications (12). Ameloblastomas arise mainly in the mandible, especially in the posterior region, of individuals between the fourth and fifth decades and do not exhibit sex predilection (12). In contrast, in the present study, ameloblastomas were slightly more common in women, with a female-to-male ratio of 1.2:1 (3).

In the present investigation, only one malignant OT was diagnosed in the elderly (1.2%), similar to previous data (3,25). Although these tumors are extremely rare at all ages, the chances of developing a malignant tumor increase with age (3,25). In addition to its rarity, which complicates the diagnosis, this group of tumors has variable biological behavior and a broad spectrum of morphological findings, which often overlap, making the morphological diagnosis challenging even for experienced pathologists. These aspects cause many odontogenic malignancies to be diagnosed only as malignant odontogenic tumors not otherwise specified (3,25).

NOCs represent only 1.15% (n = 86) of all cases diagnosed at the three centers participating in this study ( Table 1). Although these cysts are generally uncommon in the elderly, they were more frequent in our population than in a previous Taiwanese study that evaluated 7,726 oral lesions in the elderly (13). In the present study, only 0.16% (n = 13) of all diagnosed lesions were NOCs. Also, radicular cysts, ameloblastomas, and epidermoid cysts were the most prevalent lesions of each group in the elderly, respectively (13). These findings are similar to our study, except for the salivary duct cyst, the most commonly observed NOC.

According to our findings, there was only a 47.2% concordance between clinical and histopathological diagnoses in all cases, with varying levels of agreement depending on the specific lesion type (ranging from 25.4% to 53.5%, as shown in  Table 1). This low concordance has also been reported in previous studies (28-30) and highlights the importance of sending all biopsy materials for histopathological analysis to ensure an accurate diagnosis and, consequently, adequate treatment for the patient. Furthermore, it is crucial to improve diagnostic skills, regardless of clinical specialty. To achieve this goal, it is important to provide continuous personnel training and make proper use of available diagnostic tools.

In summary, more multi-institutional studies should be encouraged better to characterize the profile of oral diseases in the elderly. Also, it is crucial to highlight the importance of periodic oral examinations of older people, ideally by health professionals experienced in diagnosing oral diseases, since early diagnosis is essential to minimize patient morbidity and, consequently, improve the quality of life of these people (1), in addition to assisting in the development of public policies and prevention strategies (3,5).

Some limitations of this study need to be pointed out. First, despite the multicentric nature of the study, which involved data from three oral pathology services located in different regions of Brazil, the sample does not represent the entire Brazilian elderly population. We also consider it essential to evaluate follow-up information to determine long-term outcomes after treating these lesions. However, as this was a study carried out in oral pathology centers, unfortunately, these data were not available. In addition, we recognize as another limitation the lack of data from young individuals to compare with data from the elderly.

## Conclusions

The OCs were relatively common in the elderly, while the NOCs and OTs were rare. Overall, this population’s most common OCs, NOCs, and OTs were periapical cysts, salivary duct cysts, and ameloblastomas, respectively. Knowing the profile of the most common cystic lesions and OTs in the elderly is essential for dentists and geriatricians. Most of these lesions are asymptomatic, and early diagnosis depends on periodic clinical and radiographic examinations, helping to avoid significant morbidity and compromising the quality of life of this population.

## Figures and Tables

**Table 1:** Age group and sex distribution of cystic lesions and odontogenic tumors in older people.

Lesions	Sex (n, %)				P-value	Mean age (±SD)	Age groups				Total		P-value	Concordance between clinical and histopathological diagnosis		
	Male	Female	NI	M:F ratio			60-69	70-79	80-89	≥90	n	%		Yes	No	NI^*^
Odontogenic cysts	211	175	3	1.2:1	0.0555^§^	67.82±6.63	269	94	24	2	389	69.7	0.0011^§^	175 (53.5%)	152 (46.5%)	62
Non-odontogenic cysts	36	50	0	1:1.4		69.75±7.48	45	32	8	1	86	15.3		17 (25.4%)	50 (74.6%)	19
Odontogenic tumors	32	52	0	1:1.6		70.75±7.25	45	24	15	0	84	15.0		21 (36.8%)	36 (63.2%)	27
Total	279 (50.2%)	277 (49.8%)	3	1:1		69.44±7.12	359 (64.2%)	150 (26.8%)	47 (8.4%)	3 (0.5%)	559	100		213 (47.2%)	238 (63.2%)	108

N, number of cases; %, percentage; SD, standard deviation. For statistical analysis, the age groups were divided into Group A (60-69 years) and Group B (≥70 years). §Pearson’s chi-squared test. *Clinical diagnosis was not informed.

**Table 2:** Patient sex and anatomical site of odontogenic cysts in older people.

Odontogenic cysts	Sex						P-value	Anatomical site						P-value	Total (within the group)	
	Male		Female		NI	M:F ratio		Maxilla		Mandible		NI	Man:Max ratio			
	n	%	n	%	n			n	%	n	%	n			n	%
Inflammatory odontogenic cysts																
Periapical cyst	93	24.1	70	18.1	3	1.3:1	0.7525^§^	78	21.0	81	21.8	7	1:1	0.0008^§^	166	42.7
Residual cyst	35	9.1	33	8.5	0	1.1:1		33	8.9	30	8.1	5	1:1.1		68	17.5
Inflammatory collateral cysts	4	1.0	3	0.8	0	1.3:1		1	0.3	6	1.6	0	6:1		7	1.8
Total	132	34.2	106	27.5	3	1.2:1		112	30.2	117	31.5	15	1:1		241	62.0
Developmental odontogenic cysts																
Odontogenic keratocyst	35	9.1	33	8.5	0	1.1:1		14	3.8	52	14.0	2	3.7:1		68	17.5
Dentigerous cyst	10	2.6	9	2.3	0	1.1:1		8	2.2	9	2.4	2	1.1:1		19	4.9
Glandular odontogenic cyst	4	1.0	6	1.6	0	1:1.5		3	0.8	7	1.9	0	2.3:1		10	2.6
Calcifying odontogenic cyst	3	0.8	2	0.5	0	1.5:1		1	0.3	2	0.5	2	2:1		5	1.3
Gingival cyst of adult	2	0.5	2	0.5	0	1:1		1	0.3	3	0.8	0	3:1		4	1.0
Lateral periodontal cyst	0	0.0	3	0.8	0	0:3		1	0.3	2	0.5	0	2:1		3	0.8
Orthokeratinized odontogenic cyst	2	0.5	0	0.0	0	2:0		1	0.3	1	0.3	0	1:1		2	0.5
Odontogenic cyst not otherwise specified	23	6.0	14	3.6	0	1.6:1		15	4.0	22	5.9	0	1.5:1		37	9.5
Total	79	20.5	69	17.9	3	1.1:1		44	11.86	98	26.4	6	2.2:1		148	38.0
TOTAL	211	54.7	175	45.3	3	1.2:1		156	42.06	215	57.9	21	1.4:1		389	100

NI, not informed; N, number of cases; %, percentage. For statistical analysis, the odontogenic cysts were divided into Group A (inflammatory cysts) and Group B (developmental cysts). §Fisher’s exact test.

**Table 3:** Patient sex and anatomical site of odontogenic tumors in older people.

Odontogenic Tumors	Sex					P-value	Anatomical site						P-value	Total (within the group)	
	Male		Female		M:F ratio		Maxilla		Mandible		NI	Man:Max ratio			
	n	%	n	%			n	%	n	%	n			n	%
Benign odontogenic tumors															
Ameloblastoma	29	34.5	36	42.9	1:1.2	0.3810^§^	6	7.6	54	68.4	5	9:1	0.1772^§^	65	77.4
Odontoma^*^	1	1.2	5	6.0	1:5		2	2.5	4	5.1	0	2:1		6	7.1
Adenomatoid odontogenic tumor	1	1.2	2	2.4	1:2		1	1.3	2	2.5	0	2:1		3	3.6
Odontogenic myxoma	0	0.0	2	2.4	0:2		2	2.5	0	0.0	0	2:0		2	2.4
Calcifying epithelial odontogenic tumor	0	0.0	2	2.4	0:2		0	0.0	2	2.5	0	2:0		2	2.4
Central odontogenic fibroma	0	0.0	2	2.4	0:2		2	2.5	0	0.0	0	0:2		2	2.4
Peripheral odontogenic fibroma	0	0.0	2	2.4	0:2		0	0.0	2	2.5	0	2:0		2	2.4
Squamous odontogenic tumor	0	0.0	1	1.2	0:1		0	0.0	1	1.3	0	1:0		1	1.2
Total	31	36.9	52	61.9	1:1.7		13	16.5	65	82.3	5	5:1		83	98.8
Malignant odontogenic tumors															
Ameloblastic carcinoma	1	1.2	0	0.0	1:0		1	1.3	0	0	0	0.0		1	1.2
TOTAL	32	38.1	52	61.9	1:1.6		14	17.7	65	82.3	5	4.6:1		84	100

N, number of cases; %, percentage. For statistical analysis, the odontogenic tumors were divided into Group A (benign tumors) and Group B (malignant tumors). *Four were compound odontomas, and two were complex odontomas. §Fisher’s exact test.

**Table 4:** Patient sex and anatomical site of non-odontogenic cysts in older people.

Non-odontogenic cysts	Sex					Anatomical site (n, %)									Total (within the group)	
	Male		Female		M:F ratio	Maxilla	Mandible	Floor of the mouth	Lip	Palate	Buccal mucosa	Tongue	Nasolabial sulcus	Perioral skin		
	n	%	n	%											n	%
Salivary duct cyst	15	17.4	30	34.9	1:2	0	1	6	17	4	15	2	0	0	45	52.3
Nasopalatine duct cyst	8	9.3	7	8.1	1.1:1	15	0	0	0	0	0	0	0	0	15	17.4
Oral lymphoepithelial cyst	5	5.8	2	2.3	2.5:1	0	0	5	0	0	0	2	0	0	7	8.1
Epidermoid cyst	4	4.7	4	4.7	1:1	1	0	2	1	0	3	0	0	1	8	9.3
Dermoid cyst	4	4.7	1	1.2	4:1	0	1	2	1	0	0	0	0	1	5	5.8
Nasolabial cyst	0	0.0	4	4.7	0:4	0	0	0	0	0	0	0	4	0	4	4.7
Thyroglossal duct cyst	0	0.0	1	1.2	0:1	0	0	1	0	0	0	0	0	0	1	1.2
Bronchogenic cyst	0	0.0	1	1.2	0:1	0	0	1	0	0	0	0	0	0	1	1.2
TOTAL	36	41.9	50	58.1	1:1.4	16 (18.6%)	2 (2.3%)	17 (19.8%)	19 (22.1%)	4 (4.7%)	18 (20.9%)	4 (4.7%)	4 (4.7%)	2 (2.3%)	86	100

N, number of cases; %, percentage.

## Data Availability

The datasets used and/or analyzed during the current study are available from the corresponding author.
